# Supplementary material for: Do AutoML-Based QSAR Models Fulfill OECD Principles for Regulatory Assessment? A 5-HT1A Receptor Case
Source: Pharmaceutics. 2022 Jul 6;14(7):1415. doi: 10.3390/pharmaceutics14071415 (PMC9319483; doi:10.3390/pharmaceutics14071415)
Supplement: Supplementary file 1 [file pharmaceutics-14-01415-s001.zip › Table S3_Summary of features importance.pdf]

Table S3. Summary of features importance. av|SHAP|—mean absolute SHAP value; SMR VSA3—MOE MR VSA Descriptor 3; GATS3p—Geary autocorrelation of lag 3 weighted by polarizability; PEOE VSA2—MOE Charge VSA Descriptor 2; SaaaC—sum of aaaC; AATSC3se—averaged and centered Moreau-Broto autocorrelation of lag 3 weighted by Sanderson EN; nBondsS—number of single bonds in non-kekulized structure; AATS6dv—averaged Moreau-Broto autocorrelation of lag 6 weighted by valence electrons; GATS6p—Geary coefficient of lag 6 weighted by polarizability; PEOE VSA9—MOE Charge VSA Descriptor 9; IC2—2-ordered neighborhood information content; SLogP—Wildman-Crippen LogP; SpMAD Dzi—spectral mean absolute deviation from Barysz matrix weighted by ionization potential; JGI8—8-ordered mean topological charge; SlogP VSA1—MOE logP VSA Descriptor 1; MATS5d—Moran coefficient of lag 5 weighted by sigma electrons; ATSC5i—centered Moreau-Broto autocorrelation of lag 5 weighted by ionization potential; SpMAD Dzse—spectral mean absolute deviation from Barysz matrix weighted by Sanderson EN; GATS8se—geary coefficient of lag 8 weighted by Sanderson EN; VSA EState1—VSA EState Descriptor; SlogP VSA3—MOE logP VSA Descriptor 3; PEOE VSA8—MOE Charge VSA Descriptor 8; JGI4—4-ordered mean topological charge; SsssCH—sum of sssCH; SlogP VSA11—MOE logP VSA Descriptor 11; GATS6are—Geary coefficient of lag 6 weighted by allred-rocow EN; SdO—sum of dO; JGI9—9-ordered mean topological charge; AATS7i—averaged Moreau-Broto autocorrelation of lag 7 weighted by ionization potential; GATS7s—Geary coefficient of lag 7 weighted by intrinsic state; JGI7—7-ordered mean topological charge; CIC5—5-ordered complementary information content; Estate VSA6—EState VSA Descriptor 6; MATS7se—Moran coefficient of lag 7 weighted by Sanderson EN; VSA EState5—EState VSA Descriptor 5; nBase—basic group count; MATS7c—Moran coefficient of lag 7 weighted by Gasteiger charge; GGI8—8-ordered raw topological charge; SaasN—sum of aasN; SaasN—Geary coefficient of lag 8 weighted by intrinsic state.

**Variable**	**av|SHAP|**	**Variable**	**av|SHAP|**	**Variable**	**av|SHAP|**
SMR VSA3	0.088	SlogP VSA1	0.062	JGI9	0.041
GATS3p	0.088	MATS5d	0.061	AATS7i	0.039
PEOE VSA2	0.083	ATSC5i	0.060	GATS7s	0.039
SaaaC	0.083	SpMAD Dzse	0.059	JGI7	0.038
AATSC3se	0.082	GATS8se	0.055	CIC5	0.037
nBondsS	0.078	VSA EState1	0.053	Estate VSA6	0.033
AATS6dv	0.073	SlogP VSA3	0.052	MATS7se	0.032
GATS6p	0.071	PEOE VSA8	0.050	VSA EState5	0.032
PEOE VSA9	0.071	JGI4	0.048	nBase	0.029
IC2	0.069	SsssCH	0.048	MATS7c	0.028
SLogP	0.066	SlogP VSA11	0.046	GGI8	0.028
SpMAD Dzi	0.062	GATS6are	0.046	SaasN	0.022
JGI8	0.062	SdO	0.043	GATS8s	0.021
