# Supplementary material for: Do AutoML-Based QSAR Models Fulfill OECD Principles for Regulatory Assessment? A 5-HT1A Receptor Case
Source: Pharmaceutics. 2022 Jul 6;14(7):1415. doi: 10.3390/pharmaceutics14071415 (PMC9319483; doi:10.3390/pharmaceutics14071415)
Supplement: Supplementary file 1 [file pharmaceutics-14-01415-s001.zip › File S1_Residual_analysis_and_QQ_plot.zip.zip!/S1_Residual_analysis.pdf]

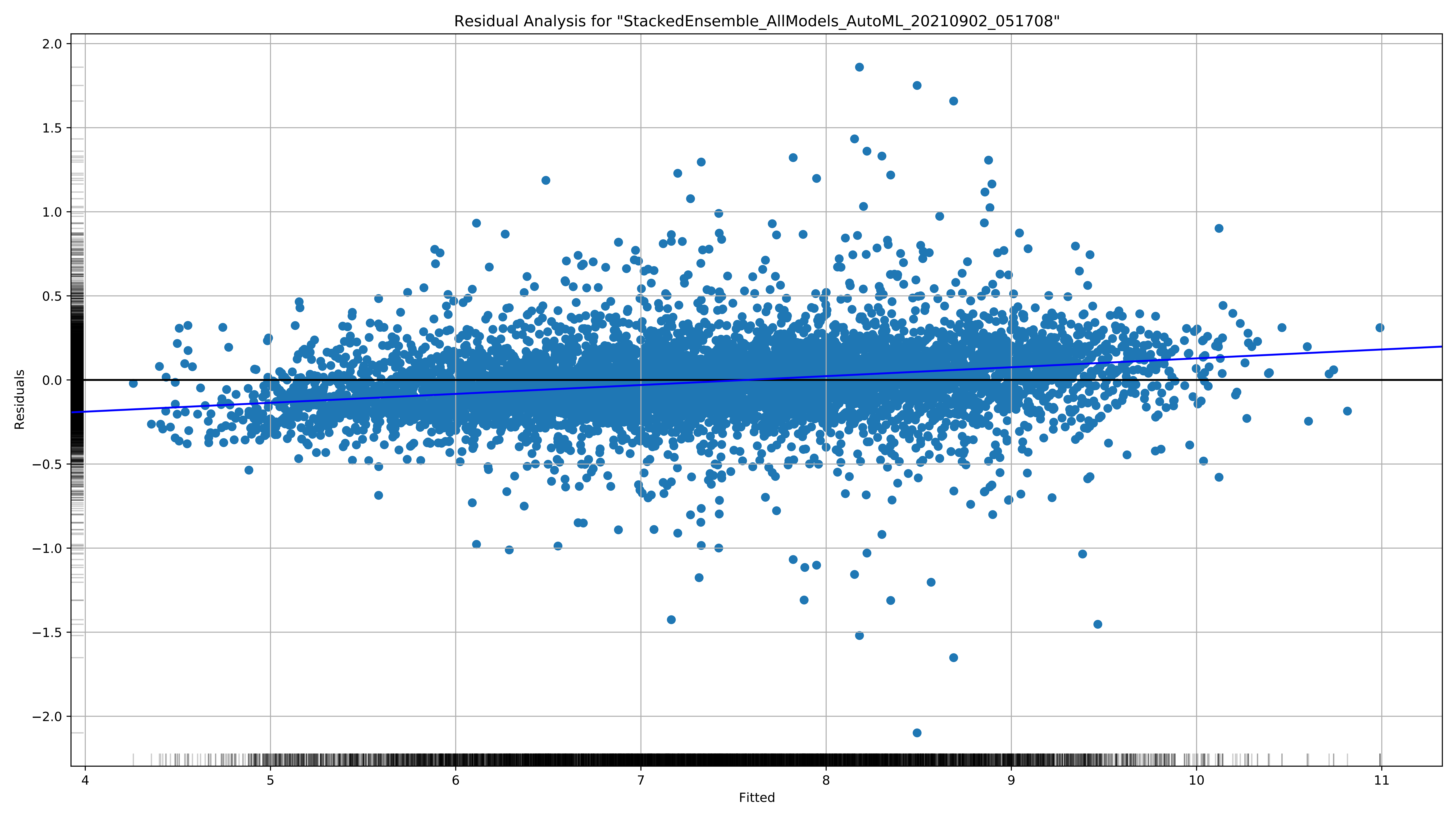

Residual Analysis for "StackedEnsemble_AllModels_AutoML_20210902_051708"
Residuals
Fitted
2.0
1.5
1.0
0.5
0.0
−0.5
−1.0
−1.5
−2.0
4
5
6
7
8
9
10
11
